# Effects of the Amylose/Amylopectin Content and Storage Conditions on Corn Starch Hydrogels Produced by High-Pressure Processing (HPP)

**DOI:** 10.3390/gels9020087

**Published:** 2023-01-19

**Authors:** Oscar Pulgarín, Dominique Larrea-Wachtendorff, Giovanna Ferrari

**Affiliations:** 1 Department of Industrial Engineering, University of Salerno, 84084 Fisciano, Italy; 2 Department of Food Engineering, Universidad del Bío-Bío, Chillán P.O. Box 447, Chile; 3 ProdAl Scarl, c/o University of Salerno, 84084 Fisciano, Italy

**Keywords:** starch-based hydrogels, amylose, amylopectin, storage conditions

## Abstract

In this study, the effects of the amylose/amylopectin content on starch gelation and the physical characteristics of hydrogels produced by HPP were studied by optical and rheological measurements in steady-state conditions. Additionally, the effects of the storage temperature (4 °C and 20 °C) and type of packaging (plastic bags or sealed Petri dishes) on the physical stability of the hydrogels were evaluated for 30 days of storage by evaluating the shrinkage of the granules, as well as the weight loss, water activity, organoleptic, and rheological properties. The experimental findings suggested that amylose plays an antagonistic role in determining the capacity of the starch granules to absorb water under pressure and to create stable and structured gels and on the physical stability of hydrogels due to its influence over the starch retrogradation extent during storage. Twenty per cent amylose was the minimum concentration to form stable corn starch HPP hydrogels with good physical and rheological properties. Moreover, a storage temperature of 20 °C and the use of polymeric bags were evaluated as the most suitable storage conditions and packaging materials enabling the long storage of corn starch hydrogels.

## 1. Introduction

Hydrogels are physical or chemical cross-linked macromolecules forming a stable three-dimensional network capable of retaining or absorbing a significant amount of water [1]. Their crosslinked network can be formed by either chemical or physical gelation processes. The gelation process based on physical methods involves physical interactions such as Van der Waals forces, electrostatic, and hydrogen bonding. For the formation of gels via chemical methods, strong chemically bonded networks must be established [2,3,4]. Hydrogels have been demonstrated as attractive tunable materials due to their peculiar properties, such as swelling in an aqueous medium, sensitivity to pH, response to temperature changes, and other stimuli [5]. Various types of hydrogels have been developed in recent years and their performance has been studied and tested in a wide range of applications, namely contact lenses [6], drug delivery systems [7,8,9], shape-memory materials [10], sensors [11], hygiene products [12], wound dressing creams [2,3], tissues engineered for medical applications [13,14], and the production of artificial muscles [15].

Innovations in the field have been recently proposed and are receiving significant attention from the scientific community, mainly consisting of the utilization of renewable and biodegradable sources, such as cellulose, lignin, pectin, and starch, as natural polymers to produce hydrogels [16]. Starch, which is a non-toxic, inexpensive, and abundantly available material, has been proven as a suitable material, economically sustainable, and appropriate for the manufacturing of natural hydrogels [9,17,18,19]. Moreover, through the years, the utilization of starch-based natural hydrogels has been proposed mainly due to their excellent body-friendly properties such as biocompatibility, hydrophilicity, and biodegradability [9,18,20,21,22].

Another recently proposed innovation consisted of the utilization of high-pressure processing (HPP) technology to achieve a sol–gel transition and obtain starch-based hydrogels. HPP is a well-known non-thermal technology consisting of subjecting a product, packaged in flexible containers, to the action of the pressure which is applied through an intermediate pressurizing medium in the range from 100 to 1000 MPa for a short time (from a few seconds to minutes) at controlled temperature conditions. Under HPP, starch-in-water suspensions experience structural and morphological changes due to the hydration and/or to the melting of crystalline structures due to compression forces and, once the threshold values of the pressure and processing time are overcome, the sol–gel transition of the system is likely to occur. On the one hand, HPP technology enabled overcoming the main limitations of the conventional gelation methods, especially a long processing time and energy consumption, and, on the other hand, obtaining hydrogels displaying good mechanical properties, stability, and a promising functionality [23,24,25]. Starch gelation via HPP depends on several factors such as the pressure level, processing time, and temperature as well as the starch source, amylose/amylopectin content, and the addition of other compounds in the formulation [23,26,27,28,29,30,31].

The ability and effectiveness of HPP to form hydrogels have been proven by utilizing different starches, namely potato, tapioca, rice, corn, and wheat starches. Regarding the amylose/amylopectin ratio characterizing starch composition, it has been shown that under HPP, the amylose content could play an important role in determining the changes in the structural characteristics and physical properties of starch granules and starch suspensions [1,32,33,34,35]. For instance, Szwengiel et al. [36] investigated the change in the molecular structure of starch granules with a different amylose content (maize starch with 0, 20.5, and 68% amylose; sorghum starch with 19.2% amylose; and amaranth starch with 0% amylose) under HPP (650 MPa for 9 min). Even though the number of α-1,4 glycoside bonds increases in pure amylopectin starches, most of the structural changes were attributed to the botanical origin of the starch. Yang et al. [34] reported that the supramolecular structure of corn starches is influenced by a different amylose/amylopectin content when subjected to high-pressure treatments. The authors have detected a conversion of waxy and normal corn starch (3.59 and 29.6% amylose content, respectively) from the A-type to B-type structure under a high pressure, but no change in high amylose-content starches (52.7 and 89.78% amylose content) has been detected. Similarly, Ji et al. [32] reported an increase in the thermal stability of corn starch suspensions treated by a high pressure when the amylose content increased. The authors hypothesize that high amylose-content corn starches could resist the high-pressure gelatinization due to the weak swelling of starch granules and the lower quantity of the amylose released. Thus, more energy should be provided to reach the complete gelatinization of these starch suspensions.

Several authors have investigated the effect of HPP on the physical properties of starch granules and starch suspensions with a different amylose content [23,24,27,30,32,35,37,38,39,40,41]; however, to the best of our knowledge, the role of the amylose/amylopectin content on the formation of hydrogels under HPP and the physical characteristics of the hydrogels eventually formed requires a further investigation. Therefore, this work aimed to study the physical and rheological characteristics of HPP hydrogels based on corn starch with a different amylose content and the stability of the hydrogels formed during a short storage period. A better understanding of the role of amylose and amylopectin in the structuring and stability of these novel materials as well as the identification of proper storage conditions of hydrogels will increase the actual knowledge of starch-based HPP hydrogels.

## 2. Results and Discussion

### 2.1. Gel Formation

The gel formation under HPP can be described as the hydration process, in excess of water, of the amorphous and crystalline regions of starch under the action of compression forces. The gelation occurrence in extent as well as the structuring level of the hydrogels formed depends on several factors, including the pressure, time, and amylose/amylopectin content, among others. The occurrence of gelatinization in starch suspensions can be assessed if the characteristic “Maltese cross” is not visible when the starch granules are observed under polarized light [31].

**Figure 1:**
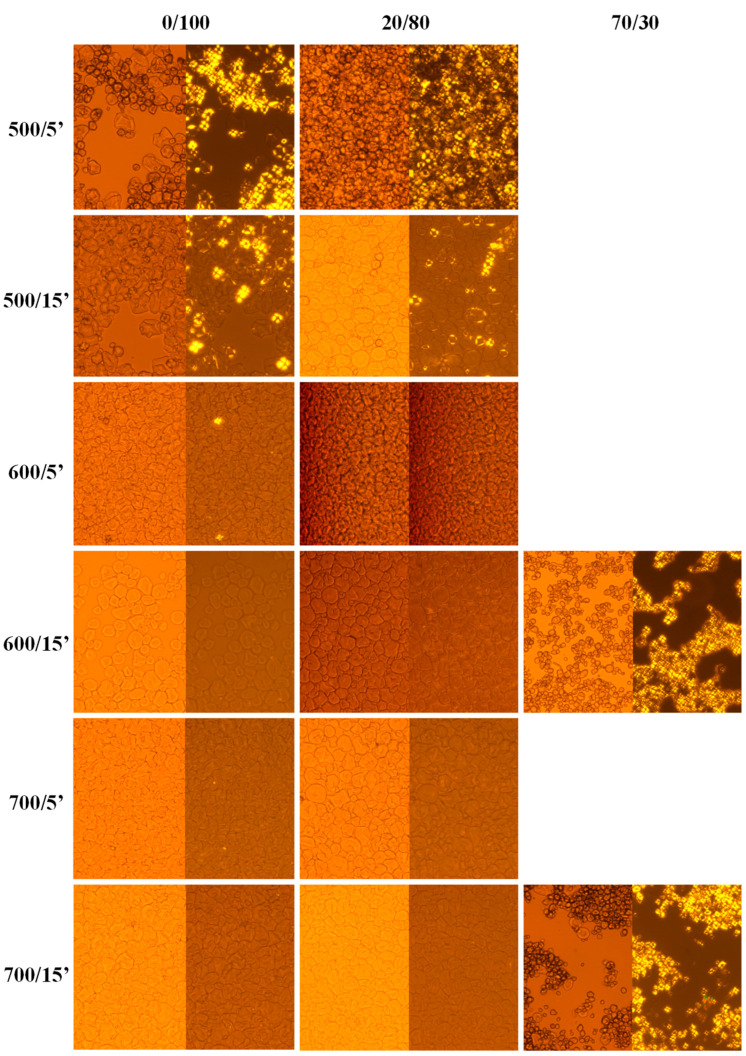
Micrographs taken under normal light (left hand side) and polarized light (right hand side, respectively) of waxy (0% amylose/100% amylopectin), normal (20% amylose/80% amylopectin), and high-amylose (70% amylose/30% amylopectin) corn starch suspensions HPP-treated at 500, 600, and 700 MPa for 5 and 15 min.

Figure 1 shows normal and polarized light micrographs of waxy corn starch (0% amylose/100% amylopectin), normal corn starch (20% amylose/80% amylopectin), and high-amylose corn starch (70% amylose/30% amylopectin) suspensions treated at 500, 600, and 700 MPa for 5 and 15 min. As can be observed in Figure 1, samples treated at 500 MPa are gelatinized only slightly, regardless of the amylose/amylopectin content. The gelatinization extent will moderately increase when the processing times are increased up to 15 min. At pressure levels of 600 and 700 MPa, waxy and normal corn starch water suspensions are completely gelatinized, which is in agreement with previous findings reported in the literature [23,27,39,40,42]. Remarkably, high-amylose corn starch suspensions were only partially gelatinized even at the most severe processing conditions applied in this work (600 and 700 MPa for 15 min). The micrographs of the samples treated at milder conditions are not shown. This result suggests that amylose at higher concentrations plays an antagonistic role in starch gelatinization by pressure, mainly due to the role played by this compound in stabilizing the crystalline structure of the starch that prevents the unwinding of the helix, thus hindering the water entrance in the granules and, therefore, starch gelatinization under HPP, as reported by Knorr et al. [43] and confirmed by Buckow et al. [40]. In addition, it is known that the chemical structure of the high-amylose corn starch is of the B-type, having a spatial configuration that hinders the production of hydrogels under viable HPP conditions, that is as high as 600 MPa, as already highlighted in papers discussing the baroresistance of this type of starch [27,35,44].

Therefore, based on the obtained results, further analyses were carried out on stable HPP hydrogels, namely those produced at 600 MPa for 5 min with normal and waxy corn starch, as described in the following.

### 2.2. Physical Characterization

Starch-based hydrogels produced by HPP are structures formed by physical non-covalent interactions of a reversible nature. Their non-permanent behavior should be examined thoroughly to understand to which extent the mechanical properties of starch-based hydrogels change over time due to water evaporation [16]. In this regard, the organoleptic properties, shrinkage index, water activity, and rheology were evaluated on HPP starch-based hydrogels packaged in plastic bags or Petri dishes immediately after HPP treatment and after 30 days of storage at 4 °C and 20 °C.

#### 2.2.1. Organoleptic Properties

**Figure 2:**
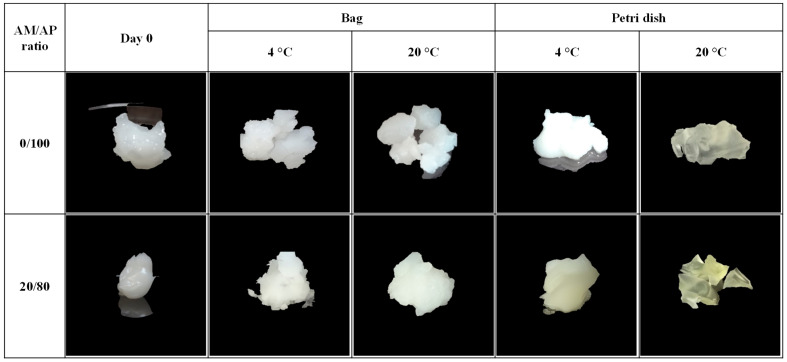
Physical appearance of hydrogels formed with starches with different amylose/amylopectin content (AM/AP ratio) at day 0 (immediately after HPP treatment) and after 30 days of storage at 4 °C and 20 °C.

As can be observed in Figure 2, the physical appearance of the corn starch hydrogels was strongly influenced by the amylose/amylopectin content. Immediately after HPP processing (day 0), all hydrogels were showing a very homogeneous structure and good integrity; the waxy corn starch hydrogels were brighter and more crystalline than normal corn starch hydrogels. These findings can be explained by considering that more superficial water can be present in HPP hydrogels based on starches containing only amylopectin molecules. As reported by Blaszczak et al. [45], granules’ swelling and the mobility of “free” and “bound” water molecules are not hindered by amylose molecules. Therefore, with an increasing amylose content in starches, the swelling of granules and the mobility of water in the gel structure decrease and, as a result, the extent of the gelatinization of starch-water suspensions decreases.

Additionally, the differences between the physical appearance of the hydrogels investigated in this work were observed during the storage period. After 30 days of storage, waxy corn starch hydrogels stored at 4 °C retained their higher brightness regardless of the type of packaging used. Normal corn starch hydrogels became drier after one month of storage. It could be hypothesized that in waxy corn starch hydrogels, water migration from the inner part of the structure to the surface causes the collapse of the structure, and then small hydrogel lumps (agglomerates) form which are mainly stabilized by non-covalent interactions such as hydrogen bonds. Xie et al. [33] reported that gelatinized waxy starches normally form clusters of “gel-balls”, especially in presence of plasticizers such as water, due to energy requirements to form more stable structures, supporting our findings.

#### 2.2.2. Shrinkage Index and Weight Loss

**Figure 3:**
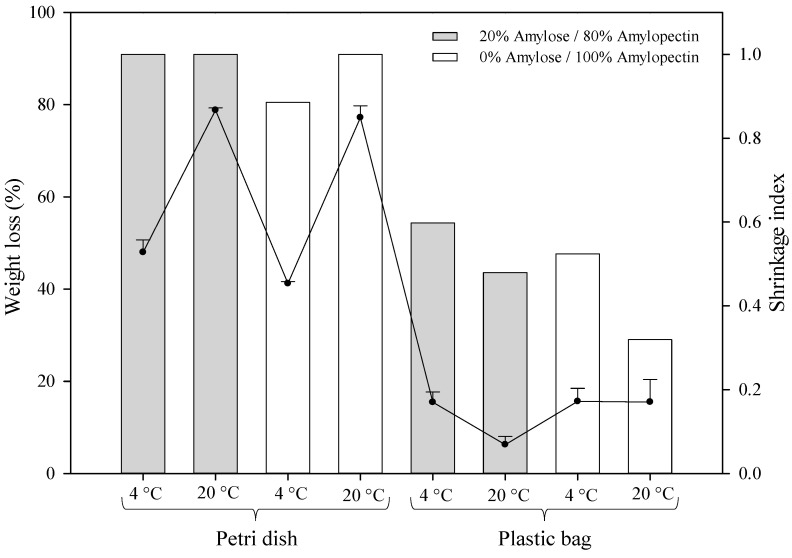
Shrinkage index (bars/right y axis) and weight loss (line/left y axis) of the different hydrogels investigated after 30 days of storage.

To better understand the role of the amylose/amylopectin content on water evaporation dynamics from starch-based HPP hydrogels, the shrinkage index and weight loss of all samples were evaluated. To this purpose, the initial granule size values and weight of all samples immediately after HPP treatment (day 0) were compared with those hydrogels at the end of the storage period (day 30) at the two different temperatures set, and the results are reported in Figure 3. It is worth noting that the shrinkage index allows for comparing the loss of water of the granules during storage with their swelling due to HPP treatment. A shrinkage index equal to one accounts for the inability of the starch granules to retain the water gained during the HPP treatment during the storage time.

From Figure 3, the shrinkage of the granules in hydrogel samples packaged in Petri dishes is more pronounced due to higher water losses, regardless of the amylose/amylopectin content of the starch. The air entering the Petri dish, even with Parafilm^®^ sealing, promotes the migration of water from the structure to the environment, meaning this mass transfer phenomenon was facilitated at higher storage temperatures (20 °C). On the contrary, hydrogels packaged in plastic bags are characterized by a lower shrinkage of the granules and a reduced weight loss (<20%). Waxy corn starch hydrogels stored at 20 °C were those showing the lowest values of the shrinkage index compared to normal corn starch hydrogels. However, a lower weight loss was detected in the latter’s structure. This interesting finding reinforces the hypothesis that amylose molecules may hinder the capacity of starch granules to absorb water during the formation of a gel under a high pressure, and even though a complete gelatinization was observed in normal corn starch hydrogels, hydrogels formed with starches composed of almost 100% amylopectin molecules entrapped more water and, therefore, more water became available to be primary released; thus, less shrinkage was likely to occur. Moreover, the shrinkage observed in both samples could be attributed to starch retrogradation phenomena. It is well known that starch gels undergo a structural reorganization of the starch chains during storage, mainly generating water loss and shrinkage, as a consequence of the crystallization of amylose and amylopectin molecules [46]. Many authors reported that the presence of amylose molecules accelerates the retrogradation of starch gels under HPP [37,47,48,49] and hinders the water absorption capacity of the starch granules by forming amylose–lipid complexes [46]. These additional observations can explain the differences observed in Figure 3.

In addition, waxy and normal corn starch hydrogels stored at 20 °C in plastic bags showed a lower shrinkage than those stored at 4 °C. This finding could be attributed to the higher retrogradation extent displayed by starch gels stored at 4 °C mainly due to the faster crystallization of the starch macromolecules, as reported elsewhere [50,51]. Recently, Li et al. [51] reported that when sago starch hydrogels were stored at room temperature, the formation of more heterogeneous and thermally stable crystallites occurred during the retrogradation process, suggesting that the gels were less prone to structural changes such as the shrinkage of granules.

#### 2.2.3. Water Activity (A_w_)

**Figure 4:**
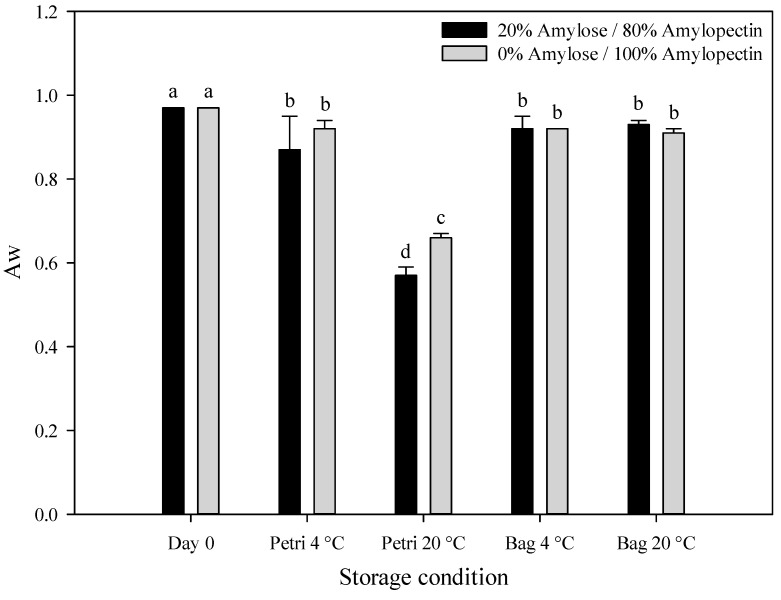
Water activity of hydrogels with different amylose/amylopectin content packed in Petri dishes and plastic bags at day 0 (immediately after HPP treatment) and after 30 days of storage at 4 °C and 20 °C. ^a–d^ Bars marked with different letters indicate significant differences (LSD, *p* ≤ 0.05).

Figure 4 reported the values of water activity of waxy and normal corn starch hydrogels immediately after the HPP treatment and after 30 days of storage. The A_w_ values of hydrogel samples were initially in the range of 0.97–0.98, regardless of the amylose/amylopectin content. Moreover, a slight decrease in the water activity was detected in all samples, except in those stored at 20 °C in a Petri dish where a drastic reduction in the A_w_ values up to 0.57–0.66 was detected. Instead, from weight loss determinations, significant differences were detected between hydrogel samples stored in plastic bags and Petri dishes. However, through a measurement of the water activity, which is a useful tool to understand water dynamics in the starch hydrogel structures, only free water on the surface of the hydrogel samples is evaluated, as reported by Larrea-Wachtendorff et al. [23]. The authors stated that water evaporation in starch-based HPP hydrogels occurred layer-by-layer from the inner core to the surface of the hydrogels; therefore, the outer surface of the hydrogels remains hydrated and the A_w_ values changed only marginally during a short storage period.

### 2.3. Rheological Properties

Rheological measurements were carried out to assess the mechanical properties of corn starch-based HPP hydrogels. In particular, the effects of the amylose/amylopectin content on the gelatinization and flow behavior of the samples were evaluated by flow measurements in steady-state conditions. Moreover, the viscoelastic properties of the hydrogels at different storage conditions were determined through frequency sweep tests within the linear viscoelastic region of the materials [23].

**Figure 5:**
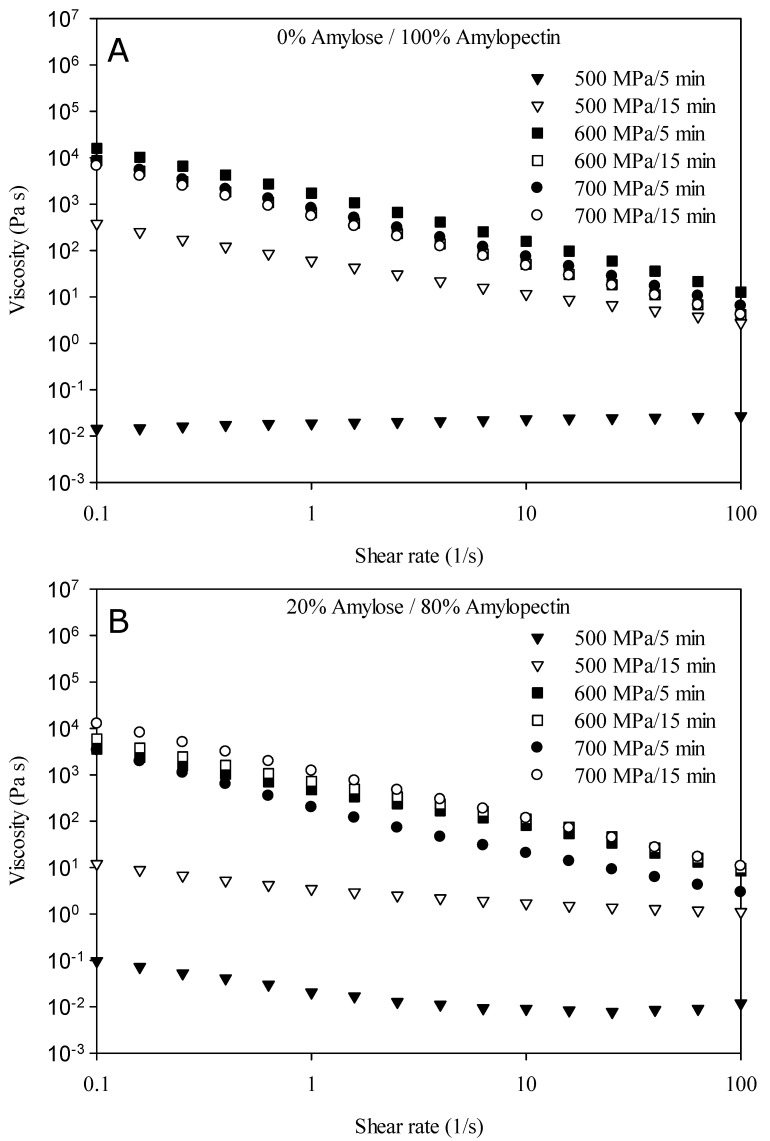
Flow behavior of corn starch HPP hydrogels with different amylose/amylopectin content, (**A**) waxy corn starch; (**B**) normal corn starch. Pressure levels: 600 and 700 MPa; treatment time: 5 and 15 min.

Figure 5 shows the viscosity of corn starch HPP hydrogels with a different amylose/amylopectin content as a function of the shear rate. The data reported only refer to starch suspensions that exhibited a gel-like structure at the processing conditions tested. In this regard, it can be noticed that corn starch suspensions treated at 600 and 700 MPa showed a gel-like structure which was less affected by the amylose/amylopectin content of the starch than by processing conditions. Indeed, both starch-based HPP hydrogels showed non-Newtonian behavior (shear-thinning), as also reported elsewhere [24,33]. They are shear-dependent flowing materials; that is, shear forces are required to cause the irreversible disruptions of the gel network, the modification of the internal structure, and the reduction in the intermolecular resistance to flow [52,53]. Moreover, it has been demonstrated that when the complete gelatinization of starch suspensions occurred, the viscosity of the hydrogels samples is affected by the different HPP processing conditions only slightly due to the higher water uptake causing the swelling of the granules and/or the higher physical interactions between the starch granules [23,24]. This could explain the similarities between the flow curves of the hydrogels formed at 600 and 700 MPa, at both the treatment times of 5 and 15 min, as reported in Figure 5.

Moreover, at 500 MPa, weak hydrogels were obtained with rheological properties far from those desired, due to the partial gelatinization extent. This confirms the results reported in Section 2.1 regarding the assessment of the formation of a gel. It is worth noting that corn starch suspensions treated at 500 MPa for 5 min were lacking any structuring level and showed Newtonian-like behavior with low viscosity values, while the suspensions treated at 500 MPa for 15 min formed a gel-like structure and showed shear-thinning behavior with higher viscosity values. This tendency was more pronounced in waxy corn starch suspensions, suggesting, again, that the amylose molecules are less prone to gelatinization by a high pressure.

**Figure 6:**
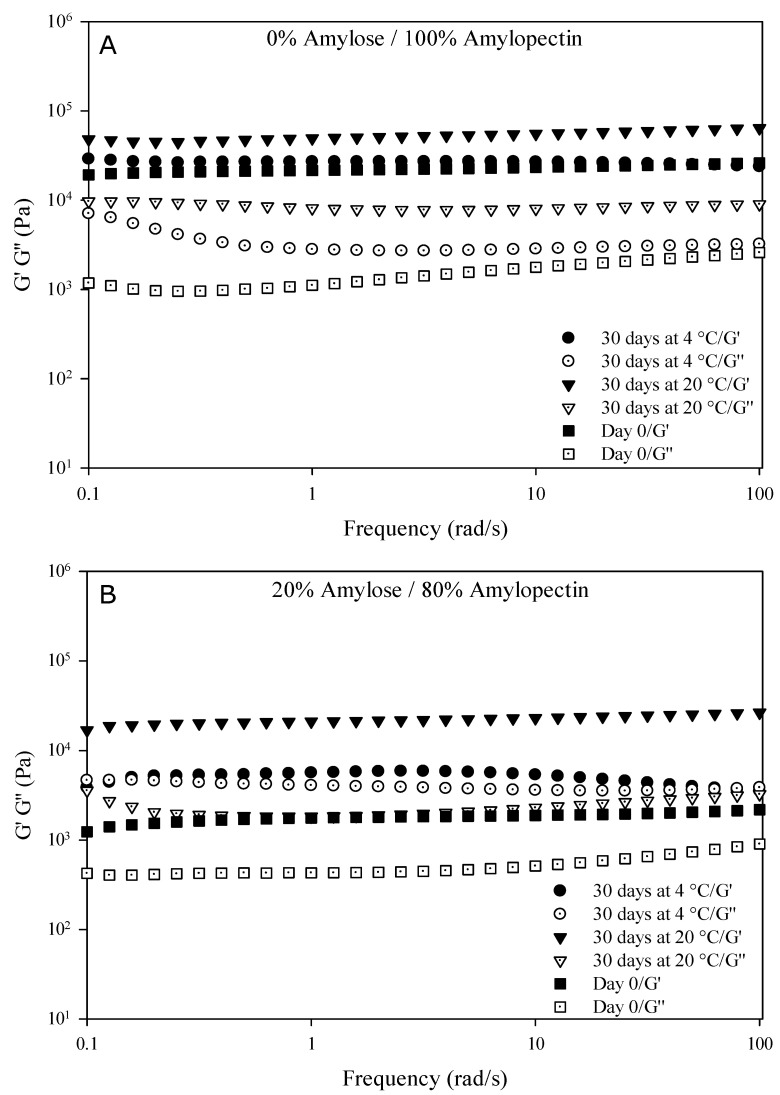
Rheology of corn starch HPP hydrogels with different amylose/amylopectin content at day 0 (immediately after HPP treatment) and after 30 days of storage in plastic bags at 4 °C and 20 °C. (**A**) waxy corn starch; (**B**) normal corn starch.

In this work, an attempt to understand the influence of the amylose/amylopectin content over the structuring level and stability of starch-based HPP hydrogels formed was also made, carrying out frequency sweep tests. They provide information on the structuring level of gels by testing the physical response of materials under controlled deformation forces. The viscoelastic properties (G’, G’’ moduli) of waxy and normal corn starch HPP hydrogels obtained at 600 MPa for 5 min were reported in Figure 6, and refer to the samples packed in plastic bags and stored for 30 days at 4 and 20 °C.

It can be observed that, initially, both starch-based hydrogels exhibited mechanical profiles characteristic of physical gels, where the elastic response (G’) of the samples prevailed on the viscous response independently of the frequency, indicating that a continuous gel network was formed between starch and water molecules [54]. Moreover, the G’ elastic response of waxy corn starch hydrogels was one order of magnitude higher than the viscous response (G’ > G’’), confirming a better structuring level and stability than normal corn starch hydrogels [55].

However, the mechanical profile of both hydrogel samples was strongly influenced by the storage time and the two temperatures investigated. After 30 days of storage, the gel network of both hydrogels was weakening, mostly related to a higher dependence of G’ and G’’ moduli from the frequency in the range investigated, and the difference between the elastic response and viscous response curves was decreasing. Higher G’ values were observed in all samples after 30 days of storage due to water evaporation and starch retrogradation. Indeed, in agreement with the results of the determination of the organoleptic properties, this trend was more marked in normal corn starch hydrogels and for samples stored at 4 °C. The G’’ values exceeded the G’ values in normal starch hydrogels stored at 4 °C; that is, a reduction in their viscoelasticity was observed. This clearly demonstrated that these samples lost their gel-like structure during storage at a low temperature. Therefore, it can be concluded that at 4 °C, starch retrogradation was likely to occur. Indeed, BeMiller [56] demonstrated that the optimal temperature for crystallization in starch retrogradation was about 5 °C, which is in agreement with our findings. Larrea-Wachtendorff et al. [57] reported that starch retrogradation is one of the main factors influencing the physical stability of starch-based HPP hydrogels during storage. Moreover, Yang et al. [34] demonstrated that starch retrogradation, which mostly depended on the role of the amylose molecules, occurred with two characteristic phenomena, namely the structural reorganization of these molecules to helix–helix conformations and their interactions with amylopectin molecules. Therefore, it can be concluded that the starch retrogradation rate and extent were higher in the samples with a higher amylose content stored at 4 °C; that is, in normal corn hydrogels rather than in waxy starch hydrogels. Starch retrogradation caused a higher gel shrinkage, water syneresis, and network weakening, as confirmed by the results reported in Figure 6, and according to the results of Yang et al. [34].

## 3. Conclusions

The experimental results obtained in this work demonstrated that the amylose content played an antagonistic role in the gelatinization by HPP treatments of corn starch water suspensions and strongly affected the physical characteristics and stability of the hydrogels obtained. At high concentrations, the amylose content reduced the swelling of the starch granules during HPP processing and lowered their capacity to retain water during storage time due to starch retrogradation. The physical and rheological changes in the hydrogels during storage were mainly attributed to the ability of the type of packaging and storage temperature to control the water evaporation and starch retrogradation. Plastic bags and a storage temperature of 20 °C were the most suitable storage conditions to preserve the physical and organoleptic characteristics of the gel-like structure of corn starch-based hydrogels during storage time (30 days). Moreover, stable corn starch HPP hydrogels with good physical and rheological properties formed when the amylose content of the starch was as high as 20%.

The results of this research represent a contribution to the advancement of knowledge on the role played by the amylose/amylopectin content in determining the structural and physical properties of starch-based hydrogels produced by HPP as a physical method for a sol–gel transition as an alternative to traditional and well-assessed gelatinization physical process. Nevertheless, further experiments should be performed to understand the behavior of starch-based HPP hydrogels in long-term storage in different conditions and to individuate natural compounds that could be added to the starch solution undergoing HPP treatments to retard or avoid starch retrogradation. The results of these experiments will be of utmost importance to individuate the optimal storage conditions for starch-based HPP hydrogels as well as their formulation to increase their stability and performances given the forecasted applicability of these novel structures in various product sectors

## 4. Materials and Methods

### 4.1. Starch-Based Hydrogels Preparation via High-Pressure Processing (HPP)

#### 4.1.1. Materials

Waxy (0% amylose-100% amylopectin, S4126), normal (20% amylose-80% amylopectin, S6425), and high-amylose (70% amylose-30% amylopectin, S6430) corn starch powders were purchased from Sigma Aldrich (Steinheim, Germany). The Amylose content of the starch samples was confirmed by an enzymatic rapid assay kit (K-AMYL, Megazyme International, Wicklow, Ireland).

#### 4.1.2. Samples Preparation

The preparation of the samples was carried out according to the method reported by Larrea-Wachtendorff et al. [25]. Briefly, starch-water suspensions (20% *w*/*w*) were formed under gentle mixing. In order to ensure sample homogeneity and avoid particles settling, the starch suspensions were prepared immediately before the HPP treatments.

#### 4.1.3. High-Pressure Processing (HPP) Treatments

The starch-water suspensions (10 g) were vacuum-packed in flexible bags (multilayer packaging OPP30/A19/LDPE70, Di Mauro 20/02/07).

The high-pressure treatments were performed using an HHP system U22 lab-scale unit (Institute of High-Pressure Physics, Polish Academy of Sciences, Unipress Equipment Division, Poland); the unit has a maximum processing volume of 50 mL and can be operated at a wide range of pressure and temperature (0–1400 MPa and 25–120 °C, respectively). The pressurizing medium is Plexol (Bis (2-Ethylhexyl) sebacate from Sigma-Aldrich, Italy) and the estimated temperature increase due to a pressure build-up is 2–3 °C/100 MPa.

After being sealed in the packages, the samples were immediately treated at pressures levels of 500, 600, and 700 MPa for 5 and 15 min at 25 °C. In Table 1, we summarized the formulation of the samples and the HPP processing conditions used in the experiments.

### 4.2. Experimental Protocol

To evaluate the stability of HPP hydrogel during a short storage time and the performance of different packaging methods to preserve their physical and organoleptic properties, the samples were stored immediately after HPP treatment for 30 days at different conditions, chilling and at room temperature, 4 °C and 20 °C, respectively, and two types of packaging, namely polymeric multilayers bags and sealed Petri dishes. In the first case, the hydrogel samples were kept in the same flexible bags used during the HPP processing of the starch solutions and they were stored at 4 °C and 20 °C. To emulate the packaging conditions of regular creams or gels, the plastic bags containing the HPP hydrogel formed were opened and the samples were aseptically poured into Petri dishes, sealed with Parafilm^®^ and stored at the above-mentioned temperature conditions. Samples at time zero (immediately after HPP processing) and the end of the storage period of 30 days were analyzed. Unless otherwise stated, all measurements were performed in triplicate.

### 4.3. Samples Analysis

#### 4.3.1. Optical Measurements

The starch granules’ optical size was determined using an optical inverted light microscope (TE 2000S, Nikon Instruments Europe B.V., Amsterdam, the Netherlands) coupled to a Camera Control Unit (DS-5M-L1, Nikon Instruments Europe B.V, Amsterdam, the Netherlands) for image acquisition and size analysis, as described elsewhere [25].

#### 4.3.2. Shrinkage Index

In order to compare the water evaporation on starch-based hydrogels during storage and the hydration of the starch granules due to HPP processing, a shrinkage index was defined, based on the change in the granules’ size during gelation and at the end of the storage period. The values of the shrinkage index were calculated according to the relationship reported in the following Equation (1):

(1) $$Shrinkage\;index=\frac{D_{d0}-D_{d30}}{D_{d0}-D_{o}}$$

*D*_0_, *D_d_*_0_, and *D_d_*_30_ are the average granule diameter of the untreated samples (corn starch in the water suspension), immediately after the HPP treatments, and after 30 days of storage, respectively.

#### 4.3.3. Weight Loss

The weight loss of samples was determined as the difference between the weight of samples at the beginning and the end of storage time. For both types of packaging, the determination was made by carefully opening them, draining any excess of water, and weighing it. The weight loss was calculated according to Equation (2):

(2) $$Weight\;loss\left(\%\right)=\frac{Hydrogel\;at\;day\;0\left(g\right)-Hydrogel\;at\;day\;30\left(g\right)}{Hydrogel\;at\;day\;0\left(g\right)}\cdot 100$$

#### 4.3.4. Visual Observation

The organoleptic properties of samples throughout the storage time were analyzed by a visual observation, making water evaporation and starch retrogradation the two phenomena that can affect the appearance, homogeneity, integrity, and color of starch-based HPP hydrogels [57]. These parameters were, thus, evaluated. In addition, the physical appearance of the samples was recorded by a digital camera (Sony Corp, Tokio, Japan) in angular mode. Original pictures without editing and filtering were reported.

#### 4.3.5. Determination of Water Activity (A_w_)

The water activity (A_w_) was determined using an AW-Sprint Novasina water activity measurement instrument (TH-500, Pfäffikon, Lachen, Switzerland). A total of 1 g of the hydrogel was poured into the center of the sample container and at least six measurements per each sample were performed to reach a hydrodynamic equilibrium inside the humidity-controlled chamber. Prior to the experiments, the water activity equipment was calibrated with standard salts at 25 °C.

#### 4.3.6. Rheology

The rheology of the hydrogels was determined in a controlled stress and strain rheometer AR2000 (TA instruments, New Castle, DE, USA) thermally controlled by a Peltier plate and a circulating water bath (DC10-Haake K10, Karlsruhe, Germany). A plate-cone geometry configuration was used (40 mm diameter, 2°, gap of 52 µm).

For the analysis, 1.0 g of the sample was loaded in the center of the rheometer plate and kept at a set temperature of 25 °C for 2 min to allow for a stress relaxation and temperature equilibration, as reported by Larrea et al. [25]. The flow curves of the samples were obtained by calculating the viscosity values at different shear rates (0.1–100 1/s). For frequency sweep tests, the viscoelastic parameters, namely the elastic modulus G’ and the loss modulus G’’, were recorded from 0.1 to 100 rad/s within the linear viscoelastic region (LVR) of the hydrogel samples, namely 3% of the strain. The LVR of the samples was determined by varying the percentage of the strain between 0.001% and 10% at a constant frequency of 0.1–1/s [23].

### 4.4. Statistical Analysis

The results were analyzed by statistical descriptive analysis (mean ± SD), a one-way ANOVA, and a post hoc comparison using Fisher’s least significant difference (LSD) test to determine significant differences amongst the experiments (*p*-value was <0.05). All analyses were performed using Statgraphics Centurion XVI Statistical Software (Statistical Graphics Corp., Herdon, VA, USA).

## Figures and Tables

**Table 1:** Samples formulation and HPP treatment conditions used in the experiments.

Amylose Content (%)	Water/ Starch (% *w*/*w*)	Applied Pressures (MPa)	Treatment Time (min)
0	80/20	500	5
20		600	
			15
70		700	

## Data Availability

The data presented in this study are available in the article.
